# Knowledge and utilisation of preconception care and associated factors among women in Ethiopia: systematic review and meta-analysis

**DOI:** 10.1186/s12978-021-01132-9

**Published:** 2021-04-15

**Authors:** Alemu Degu Ayele, Habtamu Gebrehana Belay, Bekalu Getnet Kassa, Mulugeta Dile Worke

**Affiliations:** Department of Midwifery, College of Health Sciences, Debre Tabor University, Debre Tabor, Ethiopia

**Keywords:** Knowledge, Utilisation, Preconception care, Systematic review, Meta-analysis, Ethiopia

## Abstract

**Background:**

Preconception care is the provision of biomedical, behavioural, and social health interventions provided to women and couples before conception. However, in Ethiopia, little is known and practised to support preconception care. Therefore, this study aimed to assess women’s knowledge and utilisation of preconception care and its associated factors in Ethiopia using systematic review and meta-analysis.

**Method:**

In the current meta-analysis, variables were searched from different electronic database systems, which included PubMed, Google Scholar, EMBASE, HINAR, Scopus, Web of Sciences, and Grey literature. Data were extracted using a standardised data collection measurement tool. The data were analysed by using STATA 14 statistical software. I^2^ tests assessed heterogeneity between the studies. A random-effect model was used to forecast the pooled knowledge and utilisation of preconception care.

**Results:**

Thirteen full-text studies were included. The pooled prevalence of knowledge and utilisation of preconception care among women in Ethiopia was 30.95% and 16.27% respectivelly. Secondary education (OR = 2.78, 95% CI,2.01–3.85), college and above (OR = 5.05, 95% CI,2.70–9.44), and antenatal care (OR = 3.89, 95% CI, 1.69–8.98) were significantly associated with knowledge level whereas; age (OR = 2.43, 95% CI, 1.30–4.53) and knowledge on preconception care (OR = 3.95, 95% CI,2.35–6.62) were positively associated with utilisation of preconception.

**Conclusions:**

Women’s level of knowledge and utilisation of preconception care was significantly low. Educational status and antenatal care follow-up were factors shown to affect knowledge of preconception care. Age and having a sound knowledge of preconception care indicated a significant association towards utilisation of preconception care. Thus, integrating preconception care strategies and policies that can address all the components of preconception care services with other maternal and child health services will be essential when designing effective implementation strategies to improve preconception care uptake. Besides this, advocating for better education for women, awareness creation, and increasing antenatal care services are essential.

*Prospero registration: *CRD42020218062

**Supplementary Information:**

The online version contains supplementary material available at 10.1186/s12978-021-01132-9.

## Plain english summary

Preconception care is an intervention provided to women and couples of a reproductive age irrespective of pregnancy status or desire, before conception, to improve women, newborns, and children's health outcomes.

This systematic review and meta-analysis used the following electronic database; PubMed, Google Scholar, EMBASE, HINAR, Scopus, Web of Sciences to search the primary articles. A total of 13 primary studies assessing knowledge and utilisation of preconception care (PCC) were included based on study eligibility criteria. Out of these eleven primary articles were focusing on knowledge of PCC, and six reported on utilisation of PCC.

The current systematic review and meta-analysis found that the pooled prevalence of PCC knowledge and utilisation amongst Ethiopian women was 30.95 and 16.27%, respectively. Women’s secondary education status, college and above, and history of antenatal care (ANC) follow-up was significantly associated with PCC knowledge. Whereas, women’s age and an adequate level of knowledge on PCC were found to be factors significantly associated with utilisation of PCC.

This systematic review and meta-analysis report that PCC knowledge and its utilisation amongst Ethiopian women were significantly low. Therefore, establishing, PCC strategies and policies that can address all the components of preconception care services with other maternal and child health services will be essential when designing effective implementation strategies to improve knowledge and uptake of preconception care.

## Background

Preconception care (PCC) comprises biomedical, behavioural, and social health interventions provided to women and couples before the occurrence of conception provision of information around health promotion, disease prevention, and information on treating existing diseases may assist with improving patient’s health status, and reducing behavioural and environmental factors that may contribute to poor maternal and child health outcomes [1].

PCC is essential for improving pregnancy and birth outcomes and the future health outcome of women, newborns, children, and the patient’s family [2, 3]. PCC provides an opportunity for family planning or reducing maternal and neonatal mortality and advancing long-term outcomes for adolescent girls, women, and children [4].

The Sustainable Development Goal (SDG) number three aims to reduce the global ratio of maternal mortality to less than 70 per 100,000 live births and newborn death to 12 per 1000 live births by the year 2030 [5]. PCC has a positive impact on reducing mortality and reduces the risk of adverse health outcomes for the woman, fetus, and neonates by improving the health, enhancing wellbeing, and awareness of women before scheduling and conceiving a pregnancy [6].

Even though governments, policy planners, and stakeholders are given a priority agenda for maternal and child health care services, the reduction of maternal and neonatal morbidity and mortality is not closely the target goal [7]. Preconception health care has not become part of routine practice across the globe, especially in developing countries. However, in this region, there is higher maternal, newborn, and child death. Of which 77% of maternal, newborn, child death and stillbirth could be prevented by building a platform at the community, health centre, and hospital to implement essential packages such as nutritional optimisation, preventing and treating infections, provision of family planning, screening and managing chronic medical diseases, substance abuse and lifestyle modifications [8].

Based on the World Health Organization (WHO), a recent report indicated that worldwide four out of ten women had unplanned pregnancies. As a result, 40% of pregnancies lack the necessary health interventions required before the occurrence of conception [9]. To enhance pregnancy outcomes, the Center for Disease Control (CDC) recommends risk assessment and counselling for all women of the childbearing age as a component of primary health care service [10]. However, many women globally do not have adequate access to pre-pregnancy, pregnancy, and childbirth services, mainly young, illiterate women or those residing in impoverished areas [11].

Maternal nutritional status in the preconception period has an essential role in the future life of offspring and lack of adequate nutrition in this critical period creates a transgenerational cycle of poor health outcomes. The balance of macronutrients in the maternal diet influences both birthweight and placental weight. The imbalance of protein and carbohydrate intake before and during pregnancy has been associated with reduced birth weight, gestational age, impaired glucose tolerance, and increased blood pressure in the offspring[12].

Although PCC is a crucial component of maternal and child health care services, it is not offered in many low-income countries such as Ethiopia. Hence, it has not been widely adopted because the aims and objectives are not widely understood and embraced [13, 14].

Previous studies conducted across Ethiopia, estimate a women’s knowledge and utilisation of PCC [15–27]. These studies showed that the level of a women’s knowledge on PCC ranges from 13.7% in Mekelle northern Ethiopia [24] to 63.4% [16] in west Shoa. The utilisation of PCC among women also ranges from 3.5% in Adama [27] to 38.2% in west Shewa [16]. From outcomes reported in each of the studies, there appeared to be significant variability between the level of knowledge and PCC utilisation among women in the country.

PCC is one of the critical elements for reducing child and maternal mortality and achieving other MDG goals. Women’s age, educational status, history of family planning (FP) use, previous adverse birth outcome, and chronic medical diseases have been highly correlated with levels of knowledge and utilisation of PCC in Ethiopia.

The rationale for variation in the level of knowledge and utilisation of PCC among Ethiopian women to date has not been reported. Hence, it is essential to provide evidence of the level of knowledge and utilisation and other PCC factors among women to identify existing gaps and provide recommendations around suitable strategies to increase the availability, accessibility, and utilisation of PCC in Ethiopia. This study reports on evidence of knowledge and utilisation and associated factors among women in Ethiopia.

## Methods

### Study design and protocol

This study uses a systemic review and meta-analysis approach of relevant articles on the prevalence rate of knowledge and PCC utilisation among women in Ethiopia. This systematic review was registered with the prospective international register of systematic reviews (PROSPERO, number CRD 42020218062) and was conducted following the guidelines for the Preferred Reporting Items for Systematic Review and Meta-Analyses (PRISMA)[28].

### Eligibility criteria

#### Inclusion criteria

Study participants included those women of reproductive age who resided in Ethiopia. Participants comprised women from diverse socioeconomic backgrounds, all ethnic groups, and those who spoke different dialects. This study included all published and unpublished observational (cross-sectional) studies on knowledge and utilisation of PCC and factors affecting knowledge of PCC and service utilisation amongst women of reproductive age in Ethiopia. This review included studies done until October 20, 2020, and was written in the English language.

#### Exclusion criteria

Studies were excluded: (1) if the study was not published in English and we were unable to access a copy translated in the English language; (2) qualitative papers that did not include reproductive data for women of childbearing age to be included in the analysis; (3) cases studies, as most studies lacked robust quality reproductive data to include in the analysis and to reduce the potential for identifying a patient in the review; (4) secondary works (e.g., review articles, commentaries, editorials, or dissertations/theses, conference abstracts that had not yet been published).

### Searching strategy and data source

A literature search was performed using PubMed, Google Scholar, EMBASE, HINAR, Scopus, Web of Sciences, and Grey literature. Manuscripts that report on the prevalence of knowledge and utilisation of preconception care and associated factors affecting knowledge and utilisation of PCC in Ethiopia. We also performed hand searched for cross-references to distinguish additional relevant articles.

The PECO (Population, Exposure, Comparison, and Outcomes) search format has used this review to search for pertinent studies.

#### Population

Reproductive age group women.

#### Exposure

Determinants of knowledge and utilisation of PCC (socio-demographics such as age, educational status,) reproductive related factors (history of FP, having ANC, adverse birth outcome), health care, and disease-related factor (chronic medical condition).

#### Comparison

The reported reference groups for each determinant factor in each respective study such as, knowledge of PCC among women who have attended secondary education versus those who had no formal education, and utilisation of PCC among women who had adverse pregnancy outcome versus their counterparts.

#### Outcome

Level of knowledge and utilisation of PCC among women.

Studies were searched by three authors (AD, HG, and BG) using comprehensive searching strategies. Initially, articles were searched by examining the full titles (“Knowledge, utilisation of preconception care and associated factors among women in Ethiopia”) and then keywords (“knowledge “utilisation “experience “uptake “preconception care determinants” “predictors”, “associated factors “reproductive age women, and “Ethiopia”). Besides this, studies were also searched from all included studies reference lists to find additional studies not included in our search strategies. Furthermore, to find relevant unpublished studies, Ethiopian universities’ digital libraries were searched (Addis Ababa University, University of Gondar, and Haramiya University). The searching periods were from September 20, 2020, to October 20, 2020 (Additional file 1).

### Identification and study selection

All identified studies were exported to the Endnote X7 reference manager software, and duplicated articles were excluded. Studies were screened after reading the title and abstracts. Three authors (AD, HG, and BG) screened and assessed articles independently. The studies full text was further assessed based on aims, methodology, participants/population, and critical findings (knowledge and utilisation of PCC and factors affecting knowledge and utilisation of PCC). Any disagreements were resolved through discussion and consensus-based on established criteria or through a fourth investigator (MD) if consensus could not be reached.

### Quality assessment

Each study's scientific strength and quality incorporated original cross-sectional study was assessed by using the Newcastle–Ottawa Scale quality assessment tool adapted for cross-sectional study quality assessments [29]. The tool has three core components; the tool's principal component is graded from five stars and mainly emphasises the methodological quality of each primary studies. The second components of the tool weighs-up the equivalency of the primary studies included in this systematic review and meta-analysis. The tool's last component assessed the quality of primary articles in statistical analysis and outcome point of view and was based on three stars. The qualities of each original study were weighted by three authors independently using these pointers. Those primary studies with a medium score (satisfying 50% quality evaluation criteria) and high quality ((≥ 7 out of 10) were enrolled for analysis. The three investigators' differences were managed by taking the average score of their quality evaluation outcomes (Additional file 2).

#### Data abstraction

After selecting the suitable studies selecting suitable studies, all necessary data were abstracted by three authors (AD, HG, and BG) independently using a pre-tested standardised data extraction form. This form includes primary author, year of publication, study setting, sample size, study design, response rate, the prevalence of knowledge and utilisation of PCC, and specific factors associated with knowledge and utilisation of PCC. For the second objective (factors), the information extraction format was prepared for each specific factor, i.e., women’s age, educational status, ANC visit utilisation of fertility preservation (FP), adverse pregnancy outcome, and chronic medical problem. In this study, variables were selected if two or more studies reported them as a significant factor. During discrepancy in data abstraction between the investigators, it was resolved through consensus and by a fourth investigator.

#### The outcome of interest

Level of knowledge and utilisation of PCC were the primary outcomes explored in this systematic review and meta-analysis. The second objective of the review was to determine the factors affecting the level of knowledge and utilisation of PCC.

### Publication bias and heterogeneity

Rigorous searches (electronic/database search and manual search) have been used to minimise the risk of bias. The authors' cooperative work was also critical in reducing bias, selecting articles based on the clear objectives and eligibility criteria, deciding the studies quality, and extracting and compiling the data. We examine publication bias with a visual inspection of the funnel plot graph qualitatively. Besides, Egger’s correlation tests at a 5% significant level were conducted to assess the presence of publication bias [30, 31]. Furthermore, to reduce the random variations among the primary study's point estimates, subgroup analysis was conducted by study regions and study settings. Sensitivity analysis was also performed to identifiy the potential source of heterogeneity. Heterogeneity across studies was evaluated using inverse variance (I^2^) statistics with its corresponding p-value using the random-effect model.

### Statistical analysis

We used Microsoft Excel for data entry and STATA version 14 software for analysis. The associated factors of knowledge and utilisation of PCC were examined based on eligibility criteria. We had considered at least two studies that reported on at least one associated factor of knowledge or/and utilisation in common with their measure of effect and 95% confidence interval (CI). Random effects model based on the DerSimonian-Laird method was considered to assess variations between the studies. The results were presented using texts, tables, and forest plots with measures of effect and 95% confidence interval. Statistical heterogeneity was tested via the I^2^ statistics at a *p-*value of ≤ 0.05[32].

## Result

### Description of studies

**Fig. 1:**
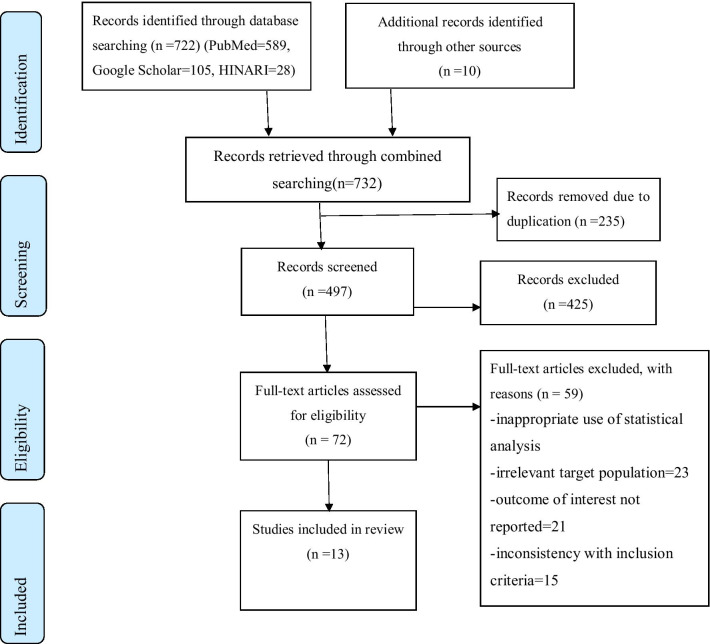
Flow chart describing the selection of studies for the systematic review and meta-analysis of the level of knowledge and utilisation of PCC and associated factors among women’s in Ethiopia, 2020

A total of 732 primary studies were identified. From these 732 identified studies, 235 were excluded after reviewing their titles due to duplication, and 497 articles were further screened for potential inclusion. Out of these 425 articles, were excluded due to irrelevance and 59 were removed due to inappropriate use of statistical analysis, irrelevant target population, inconsistent study report. Thirteen articles fulfilled the inclusion criteria and were included in this systematic review and meta-analysis with a total population of 6,904 women (Fig. 1).

### Characteristics of the included studies

These 13 eligible studies were cross-sectional in design and were reported in the English language. The sample size ranged from 142 women who reside in Addis Ababa [19] to 680 Western Shoa zone of Oromia [16]. Regarding geographical distribution of studies, four studies were from Oromia (30.7%) [16, 18, 23, 27], four from Amhara (30.7%) [15, 22, 25, 26], two from Tigray (15.4%) [21, 24], two from (15.4%) SNNP[17, 20] and one from AA (7.8%) [19]. Eleven of the included studies reported on knowledge[15–25], and six provided data on utilization of PCC[16, 18, 22, 24, 26, 27] (Table 1). Once more, out of the thirteen included studies, eight had a quality score of eight, and the remaining five had a quality score of seven. Hence, all of them had moderate quality (Additional file 2).

**Table 1:** Summary of the 13 observational studies included in the meta-analysis assessing women level of knowledge and utilisation of PCC in Ethiopia, 2020

Author’s, year	Study period	Study setting	Study population	Sample size	Parameter studied	Prevalence (%) with 95% CI
Ayalew et al. (2017) [15]	2016	Community	Reproductive age women	422	Knowledge of PCC	27.5(25.5–29.4)
Fekene et al. (2020) [18]	2017	Community	Reproductive age women	680	Knowledge and Utilisation of PCC	26.8(24.9–28.7) 14.9(13.3–16.4)
Teshome et al. (2020) [23]	2019	Community	Pregnant women	636	Knowledge of PCC	21.3(19.5–23.0)
Yohannes et al. (2019) [20]	2017	Institutional	Delivered women	373	Knowledge of PCC	53(50.8–55.1)
Andualem [Unpublished]	2016	Institutional	Pregnant women	634	Knowledge and utilisation of PCC	63.4(61.3–65.5) 38.236.0–40.3()
Kassie (Unpublished)	2018	Institutional	Pregnant with diabetic	143	Knowledge PCC	42.7(40.5–44.9)
Kassa and Yohannes [17]	2017	Institutional	Pregnant women	580	Knowledge PCC	20(18.2–21.7)
Demssie et al. (2019) [22]	2017	Community	Reproductive age women	424	Knowledge and utilisation PCC	17.3(15.6–18.9) 13.4(11.9–14.9)
Abrha et al. (2020) [21]	2018	Community	Delivered women	564	Knowledge PCC	39(36.9–41.1)
Assresu et al. (2019) [24]	2018	Community	Delivered women	564	Knowledge and utilisation PCC	13.7(12.2–15.2) 18.2(16.5–19.9)
Goshu et al. (2018) [25]	2016	Community	Reproductive age women	422	Knowledge of PCC	15.9(14.3–17.5)
Dessie et al. (2018) [27]	2014	Institutional	Pregnant women	422	Utilisation of PCC	3.5(2.7–4.3)
Goshu et al. (2018) [25]	2016	Community	Pregnant women	229	Utilisation of PCC	9.6(8.3–10.9)

### Knowledge and utilisation of preconception care

**Fig. 2:**
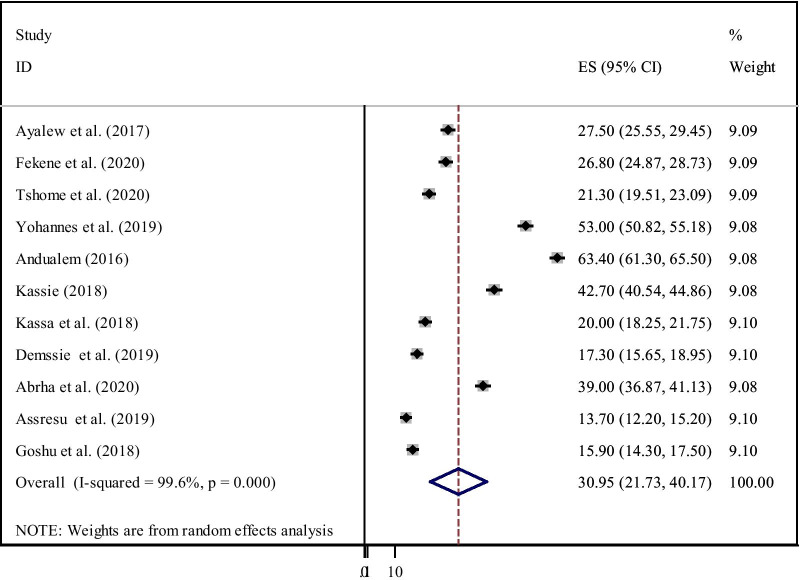
Forest plot of the pooled prevalence of women’s knowledge on preconception care in Ethiopia, 2020

**Fig. 3:**
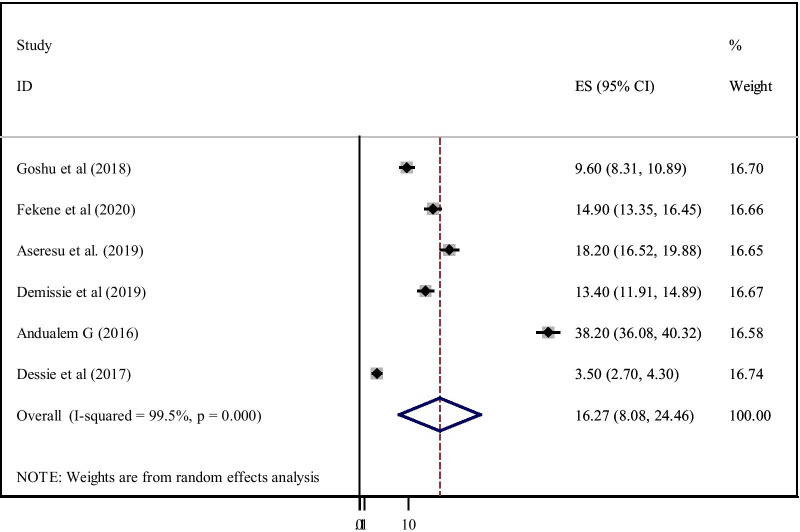
Forest plot of the pooled prevalence of utilisation of PCC of women in Ethiopia,2020

The overall pooled prevalence of an acceptable level of PCC knowledge among women in Ethiopia was 30.95% (95% CI:21.73–40.17) (Fig. 2). The pooled prevalence of PCC utilisation in Ethiopia was 16.27% (95% CI: 8.08–24.46) (Fig. 3).

### Heterogeneity and publication bias

Among the 13 studies eligible for meta-analysis, eleven studies dealing with PCC knowledge showed high heterogeneity using the I^2^ test (I^2^ = 99.7%, P ≤ 0.001), indicating random-effects modelling. Six studies included in this meta-analysis to pool PCC utilisation also showed high heterogeneity as evidenced by the I^2^ test (I^2^ = 99.5%, P ≤ *0.001*).

**Fig. 4:**
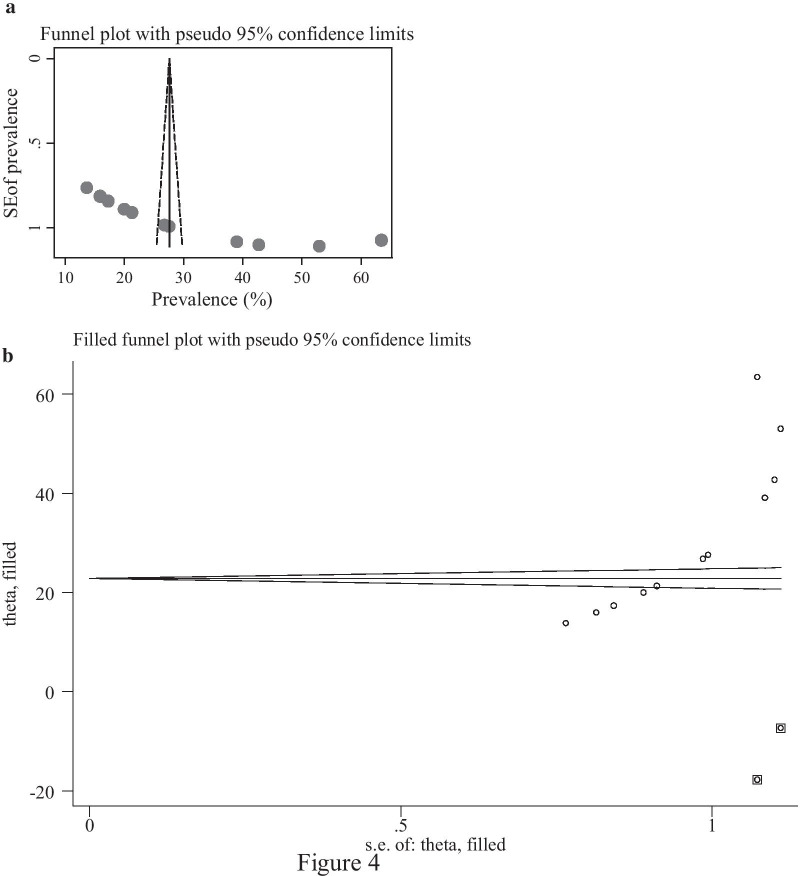
**a** Funnel plot to test the publication bias of 12 studies. **b** Result of trim and fill analysis for adjusting publication bias of the 13 studies

**Fig. 5:**
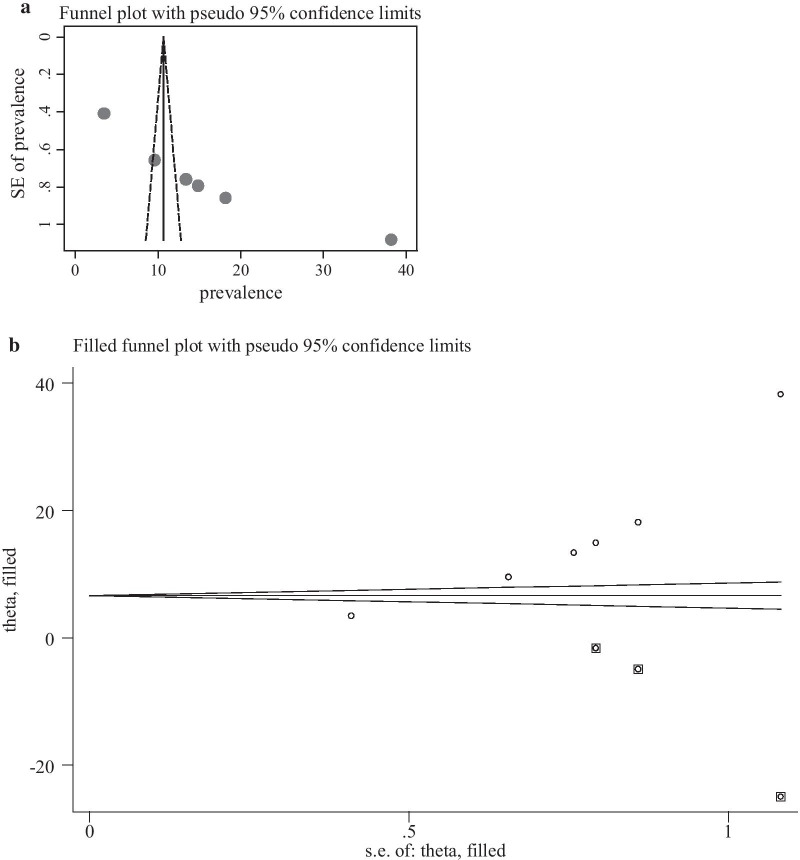
**a** Funnel plot to test the publication bias of 6 studies. **b** Result of trim and fill analysis for adjusting publication bias of the nine studies

Publication biases among the 11 included studies to assess the pooled knowledge were examined using funnel plots and Egger’s regression test. The funnel plots showed an asymmetric shape, which indicated the presence of publication bias (Fig. 4a). Egger’s regression test also demonstrated the existence of publication bias across studies (p-value < 0.001). The Duval and Tweedie non-parametric trim and fill analysis was conducted to correct publication bias among the 11 studies reporting on PCC knowledge. Moreover, publication bias was corrected when two missed studies were filled in the funnel plot by trim and fill analysis. After two studies were filled, a total of 13 studies were included and computed via the trim and fill analysis to produce the pooled prevalence of 24.26% (95% CI, 13.39–35.13) by applying a random-effects model (Fig. 4b). Publication bias was also observed among six studies eligible for assessing utilisation of PCC as evidenced by the asymmetrical shape of the funnel plot (Fig. 5a) and Eggers regression test (P < 0/001). A trim and fill analysis was also conducted on six studies reporting PCC utilisation, and publication bias was corrected when three missed studies were filled. After a total of nine studies were included and computed through the trim and fill analysis, a pooled prevalence of 7.36(95% CI: 1.09–15.82) was highlighted by using the random-effect model (Fig. 5b).

### Sensitivity analysis

In the current meta-analysis, to determine the potential source of heterogeneity seen in the pooled prevalence of PCC knowledge, the investigators performed a leave-one-out sensitivity analysis. The result of the sensitivity analysis indicated that the finding did not rely on a particular study. The pooled prevalence of level of knowledge of PCC was varied and ranged from 27.70% (20.14–35.26%) to 32.67% (22.98–42.37%) after deletion of two studies (Table 2).

**Table 2:** Sensitivity analysis of the prevalence of level knowledge and PCC utilisation among women in Ethiopia, 2020

Study omitted	Prevalence (%)	95% CI
For knowledge of PCC		
Ayalew et al. [15]	31.29	21.15–41.43
Fekene et al. [18]	31.36	21.78–41.51
Teshome et al. [23]	30.10	21.78–42.04
Yohannes et al. [20]	28.74	19.78–37.70
Andualem [Unpublished]	27.70	20.14–35.26
Kassie [Unpublished]	29.77	20.10–39.45
Kassa and Yohannes [17]	32.04	21.94–42.14
Demssie et al. [22]	32.31	22.31–42.31
Abrha et al. [21]	30.14	20.30–39.99
Assresu et al. [24]	32.67	22.98–42.37
Goshu et al. [25]	32.45	22.54–42.36
For utilization of PCC		
Goshu et al. [25]	17.61	7.27–27.95
Fekene et al. [18]	16.54	6.79–26.29
Assresu et al. [24]	15.88	6.52–25.24
Demssie et al. [22]	16.84	6.92–26.77
Andualem [Unpublished]	11.89	6.23–17.55
Dessie et al. [27]	18.83	10.64–27.02

### Subgroup analysis

Sub-group analysis was performed based on the region where the primary studies dealing with PCC knowledge were conducted. Consequently, the highest pooled prevalence was observed in Oromia National regional state ( 37.16% (95%CI, 12.22–62.10)), and the lowest pooled prevalence was reported in Amhara National regional state (20.21% (95%CI, 13.50–26.93)). Furthermore, the sub-group analysis was stratified by study setting, indicating that institutional-based studies had a higher pooled prevalence of 44.77(95% CI,25.24%-64.30) than community-based studies 23.05% (95%CI, 16.95–29.14). The sub-group analysis of the utilisation of PCC by region was higher in Oromia with a pooled prevalence of 18.84% (95%CI, 1.80–36.88) and lowest in Amhara with a pooled prevalence of 20.21% (95%CI, 13.50–26.93) (Table 3).

**Table 3:** Subgroup analysis of knowledge and utilisation of PCC among women in Ethiopia, 2020

Variables	Characteristics	Included studies	Number of study participants	Prevalence (95% CI)	*I*^2^(%), *P-value*
For knowledge of PCC					
Region	Amhara	3	1268	20.21 (13.50–26.93)	97.8 < 0.001
	Oromia	3	1950	37.16 (12.22–62.10)	99.8 < 0.001
	SNNP	2	954	36.49 (4.15–68.83)	99.8 < 0.001
	Addis Ababa	1	142	42.70 (40.54–44.86)	–
	Tigray	2	1128	26.34 (1.54–51.13)	99.7 < 0.001
Study setting	Community	7	3712	23.05 (16.95–29.14)	98.8 < 0.001
	Institutional	4	1731	44.77 (25.24–64.30)	99.7 < 0.001
Overall		11	5443		
For utilization of PCC					
Region	Amhara	2	653	11.48 (7.76–15.20)	93.0 < 0.001
	Oromo	3	1736	18.84 (1.8–36.88)	99.8 < 0.001
	Tigray	1	564	18.20 (16.52–19.88)	–
Overall		6	2953	16.27 (8.08–24.46)	99.5 < 0.001

### Factors of knowledge about PCC

**Fig. 6:**
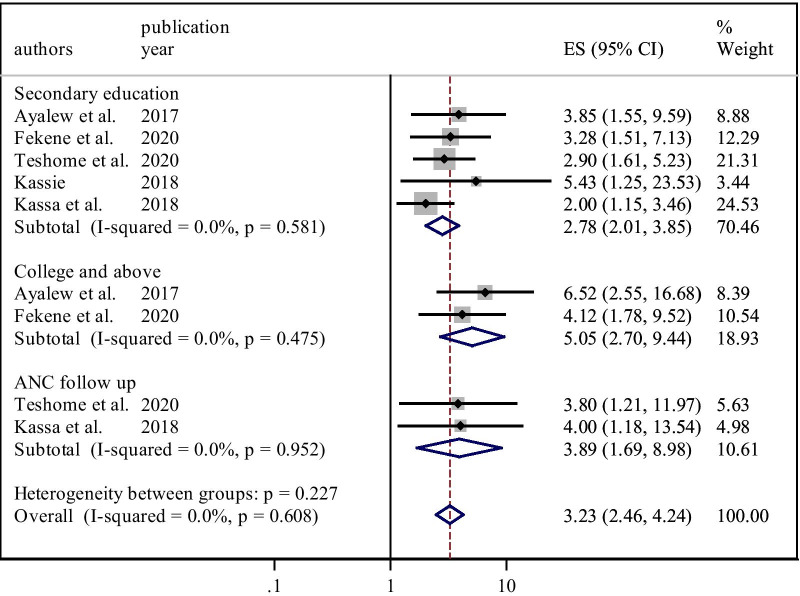
Factors affecting women’s knowledge of PCC in Ethiopia,2020

This systematic review and meta-analysis revealed that PCC knowledge amongst women in Ethiopia was significantly associated with educational status, FP history, and ANC visit. According to this study, five primary articles identified that secondary education was significantly associated with PCC knowledge [15, 17–19, 23]. Women who attended secondary education were nearly three times (OR = 2.78, 95% CI, 2.01–3.85) more likely to know about PCC than women who had no formal education. The heterogeneity test indicated I^2^ = 0.0%, *P* = *0.58* hence the fixed-effect model was applicable for analysis (Fig. 6).

Furthermore, two studies also reported that college and above educational status were strongly associated with PCC knowledge[15, 18]. Compared with those who had no formal education, women who attended college and above were five times (OR = 5.05, 95% CI,2.70–9.44) more likely to know about PCC. The heterogeneity test showed that I^2^ = 0.0%, *P* = *0.47* hence the fixed-effect model was used for analysis (Fig. 6).

This systematic review and meta-analysis concluded that having an ANC visit was a significant predictor of PCC knowledge, as highlighted in two studies [17, 23]. Women who attended at least one ANC visit had nearly four times (OR = 3.89, 95% CI, 1.69–8.98) higher odds to receive an adequate level of knowledge on PCC than counterparts. The heterogeneity test showed an I^2^ value of 0.0%, *P* = *0.95* hence, we used the fixed-effect model for analysis (Fig. 6).

### Factors affecting the utilisation of PCC

**Fig. 7:**
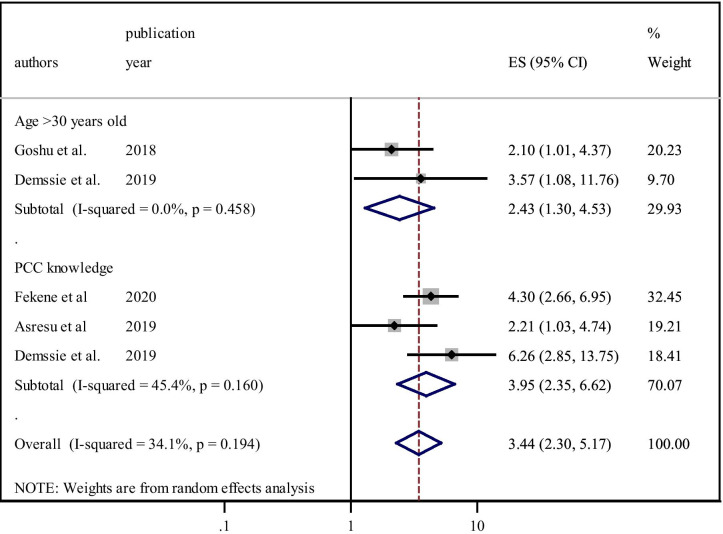
Factors affecting utilisation of PCC in Ethiopia, 2020

This study showed that the utilisation of PCC among women in Ethiopia was significantly associated with age (> 30 years) and knowledge of PCC. According to this meta-analysis, two primary studies identified that older age (> 30 years) was significantly associated with the utilisation of PCC [22, 26]. Those women who were older (> 30 years) were approximately two and a half times (OR = 2.43, 95%CI, 1.30–4.53) higher concerning utilising PCC compared to their counterparts. Heterogeneity was not observed across the studies (I^2^ = 0.0%, P = 0.45) (Fig. 7).

Three studies revealed that having adequate knowledge about PCC was strongly associated with utilisation [18, 22, 24]. Women who were knowledgeable about PCC had nearly four times (OR = 3.95, 95%CI,2.35–6.62) more likely to engage in service utilisation than their counterparts. The heterogeneity test showed that I^2^ = 45.4%, *P* = *0.16* hence, the random-effect model was used for analysis (Fig. 7).

## Discussion

This systematic review and meta-analysis reports about the level of knowledge and utilisation of PCC. The evidence was summarised in terms of the level of knowledge and utilisation of PCC among women who resided in Ethiopia. Accordingly, the pooled prevalence of an adequate level of knowledge and utilisation of PCC among Ethiopian women was 30.95% (95% CI:21.73–40.17) and 16.27% (95% CI: 8.08–24.46), respectively. PCC can address a full range of women’s and couples' health needs to support women’s pregnancy and the fetus and neonates health and welfare. According to this meta-analysis results, the pooled prevalence of a good level of knowledge about PCC was 30.95% (95% CI:21.73–40.17).

Even though PCC could address a full range of women’s, and couple’s health needs to produce healthy newborn, family and a community at large, there was no analogous meta-analysis study conducted on this specific research question within the area. The pooled prevalence of the level of knowledge of PCC among women is consistent with other studies conducted in Utah (32%) [33], Saudi Arabia (38.9%) [34], China (34.6%) [35], and Kenya (37.3%) [36]. However, it is lower than the survey studies carried out in China (90%) [37] Qatar (53.7%) [38], Canada (70%) [39], British Colombia (71%) [40], Turkey (46.3%) [41], USA (76%) [42], and the United Arab Emirates (46.4%) [43]. The low level of knowledge reported in this study might be due to the low socio-economic status, the discrepancy in the infrastructure of the health sectors, lack of promotion of PCC reported in the media, the insufficient attention given to PCC implementation by the health care system across the country, and lack of preconception clinic at the health institution level, and low commitment of health care workers due to high case flow of the patient/clients. However, outcomes reported in this study are higher than those reported in studies from Nepal 15.42% [44], Iran 10.4% [45], and Iraq 3.3% [46]. This may be due to time variation, and attention given to the discipline of maternal health in Ethiopia, which may result in an overall increment in the knowledge of maternal health. The discrepancies observed may also account for contextual differences in study settings. This finding implies that the awareness creation activities/strategies of PCC were inadequate; however, they require further research to boost women’s knowledge. Therefore, there is a need to involve multiple stakeholders, governmental organisations, and non-governmental organisations in PCC.

This meta-analysis also attempted to determine the pooled prevalence of PCC uptake; 16.27% (95%CI: 8.08–24.46) of Ethiopian women utilised at least one PCC component. These findings are The finding is in line with studies reported from China 20.6% [47],London 27% [48], Maryland 33.1% [49], and Brazil 15.9% [50]. However, results are lower than survey studies conducted in China 40% [37], Canada 44% [51], Surveillance Summaries of CDC 74.9% [52], Los Angeles 28.8% [53], USA 35% [54], and British Colombia 49.4% [40]. Findings reported might be due to the difference in socio-demographic status, study setting, study participants, and healthcare system in these countries. It is also explained by poor policies and guidelines and low media coverage for PCC in Ethiopia. On the other hand, outcomes reported in this study are higher compared with those studies conducted in Sudan at 9% [55] and Brazil at 7.9% [56]. Variability may be related to differences in each country’s study population’s education, culture, and study setting.

Women who had secondary education were nearly three times more likely to be knowledgeable than women who had no formal education. Findings were comparable to outcomes illustrated in studies from Sudan [55], Nigeria [57], and Iran [58]. Finding may be explained as the women’s educational status increases; their health-seeking behaviour regarding PCC will also improve. Hence, those women with higher education levels might be eager to know about their health status, and risk factors leading to ill health, hence poorer maternal and reproductive outcomes. Also, more educated women might have greater access or be more inclined to acquire information sources in relation to their wellbeing and have better reproductive health management plans.

Similarly, women who attended college and above were five times more likely to have increased knowledge than those with no formal education. The findings presented were in parallel with studies conducted in Nigeria [59], Sudan [55], Iran [45] USA [42], Sri Lanka [60], and the Netherlands [61]. The findings presented might also be due to the higher educational level having greater access to reproductive health information. Furthermore, technologies such as the internet provide extensive information. Besides, educated women had a broader understanding of complications associated with not using PCC. Moreover, education improves communication with the partner, women’s status in the community, and the influence of education on women’s decision-making skills to access appropriate information.

The current meta-analysis also revealed that knowledge scores increase with the frequency of ANC visits. Women who had at least one or/and more ANC visits presented with good knowledge scores on PCC. Our findings were similar to those conducted in Sudan [62], and Nigeria [63]. One potential rationale might be due to ANC visits. Hence, women may be provided with an opportunity to speak with their healthcare provider about questions and concerns that they may have concerning their reproductive health and provide their health care provider with details surrounding their gynaecological and obstetric well detailed family medical history. During an ANC visit, women can also get information on their health status, information on disease prevention and health promotion, and birth preparedness and discuss potential obstetric complications that they may need to know, such as vaginal bleeding, severe headache, epigastric pain blurring of vision.

In this meta-analysis women whose age was greater than 30 years of age were 2.4 times more likely to utilise PCC than younger women. Similarly, studies conducted in Brazil [50], Okhlama [64], and USA [65] showed that older women were more likely to utilise PCC services and attend ANC visits compared with younger women. This finding might be because older women may have believed they were not at an ideal age for childbirth and are at risk of developing pregnancy complications. As a result, they tended to use PCC.

Lastly, our study reports that women’s knowledge was positively associated with PCC utilisation.

Those women who had good knowledge of PCC services were nearly four times more likely to utilise PCC services than those with inadequate knowledge. Findings from China [66], Saudi Arabia [34], France [67], Philadelphia [68], and Nigeria [63] provided similar outcomes to those reported in this study. Women with some basic level of knowledge were more aware of costs associated with not using PCC services.

Moreover, comprehensive knowledge of PCC may provide better insight and awareness, which can positively impact the overall healthy life of women, newborns, and the wider-community.

Despite its significant importance, this systematic review and meta-analysis have limitations. First of all, all of the studies included in this review were cross-sectional; hence, the outcome variable might be influenced by other confounding variables such as misconceptions, knowledge, accessibility of the care. Second, some of the studies included in this systematic review and meta-analysis had small sample sizes, which may affect the actual magnitude of knowledge and utilisation at the country level. Third, all Ethiopia regions were not represented in this systematic review and meta-analysis due to a limited number of studies in the country (only four regions and one administrative town were represented in this study).

## Conclusions

Our findings presented in this study showed that women’s level of knowledge and utilisation of PCC was significantly low. Educational status and ANC follow-up were factors that affect knowledge of PCC and older age and having sound knowledge of PCC had shown significant association towards utilisation of PCC. Thus, integrating PCC strategies and policies that can address all the components of preconception care services with other maternal and child health services will be essential when designing effective implementation strategies to improve preconception care uptake. Besides this, advocating for better education for women, awareness creation, and increasing antenatal care services are essential. Furthermore, we recommend synchronised and cooperated work between the clinical and research teams to change knowledge and utilisation of PCC services.

## Supplementary Information

**Additional file 1.** A searching strategy for knowledge and utilisation of PCC and associated factors among women in Ethiopia.

**Additional file 2.** Newcastle–Ottawa Quality Assessment Scale for cross-sectional studies to assess knowledge and utilisation of PCC among women in Ethiopia.

## Data Availability

The data that support the review findings of this study are available upon a reasonable request to the corresponding author.

## Abbreviations

ANC: Ante-Natal Care
PCC: Preconception care
