# Supplementary material for: Knowledge and utilisation of preconception care and associated factors among women in Ethiopia: systematic review and meta-analysis
Source: Reprod Health. 2021 Apr 15;18:78. doi: 10.1186/s12978-021-01132-9 (PMC8048176; doi:10.1186/s12978-021-01132-9)
Supplement: Supplementary file 1 — Additional file 1. A searching strategy for knowledge and utilisation of PCC and associated factors among women in Ethiopia. [file 12978_2021_1132_MOESM1_ESM.docx]

**Additional file 1:** Searching strategy for knowledge and utilisation of PCC and associated factors among women in Ethiopia,2020.

| Databases | Searching terms | Number of studies |
| --- | --- | --- |
| PubMed | “Knowledge” [MeSH Terms] OR “knowledge” [All Fields] OR “preconception care uptake”[MeSH Terms] OR “preconception care utilization”[All Fields] OR “preconception care”[All Fields] OR “preconception care use”[MeSH Terms] OR “preconception care use”[All Fields]) AND “associated factors”[MeSH Terms] OR “determinants” [All Fields] AND “among women”[All Fields] OR “reproductive age women”[MeSH Terms] AND “Ethiopia” [MeSH Terms] | 589 |
| Google scholar | "Knowledge” AND "utilization” AND "preconception care” AND “associated factors” AND “women” AND “Ethiopia" | 105 |
| HINARI | “Knowledge” AND “utilization” AND “preconception care” AND “associated factors” OR “determinants” OR “predictors” AND “women” AND “Ethiopia” | 28 |
| Others databases |  | 10 |
| Total retrieved |  | 732 |
| Included |  | 13 |
