# Supplementary material for: Knowledge and utilisation of preconception care and associated factors among women in Ethiopia: systematic review and meta-analysis
Source: Reprod Health. 2021 Apr 15;18:78. doi: 10.1186/s12978-021-01132-9 (PMC8048176; doi:10.1186/s12978-021-01132-9)
Supplement: Supplementary file 2 — Additional file 2. Newcastle–Ottawa Quality Assessment Scale for cross-sectional studies to assess knowledge and utilisation of PCC among women in Ethiopia. [file 12978_2021_1132_MOESM2_ESM.docx]

**Additional file 2:** Newcastle-Ottawa Quality Assessment Scale for cross sectional studies to assess knowledge and utilisation of PCC among women in Ethiopia,2020.

| Authors | Representatives | Sample size | None-responders | Ascertainment | comparability | outcome | Quality score |
| --- | --- | --- | --- | --- | --- | --- | --- |
| Ayalew et al. (2017) | 1 | 2 | 1 | 1 | 1 | 1 | 7 |
| Fekene et al. (2020) | 1 | 2 | 1 | 2 | 1 | 1 | 8 |
| Teshome et al. (2020) | 2 | 1 | 1 | 2 | 1 | 1 | 8 |
| Yohannes et al. (2019) | 1 | 1 | 1 | 2 | 1 | 1 | 8 |
| Andualem(unpublished) | 2 | 1 | 1 | 1 | 1 | 1 | 7 |
| Kassie (unpublished) | 1 | 1 | 1 | 1 | 2 | 1 | 7 |
| Kassa et al. (2018) | 1 | 1 | 1 | 2 | 1 | 1 | 8 |
| Demssie et al. (2019) | 1 | 2 | 1 | 1 | 2 | 1 | 8 |
| Abrha et al. (2020) | 2 | 1 | 1 | 1 | 2 | 1 | 8 |
| Assresu et al. (2019) | 1 | 2 | 1 | 1 | 2 | 1 | 8 |
| Goshu et al. (2018) | 1 | 1 | 1 | 1 | 2 | 1 | 7 |
| Dessie et al. (2018) | 1 | 2 | 1 | 1 | 2 | 1 | 8 |
| Goshu et al. (2018) | 2 | 2 | 1 | 1 | 1 | 1 | 7 |

Interpretation of the score

Very Good Studies: 9-10 points

Good Studies: 7-8 points

Satisfactory Studies: 5-6 points

Unsatisfactory Studies: 0 to 4 points
